# Temporal Incidence of Eriophyid Mites on Rose Rosette Disease-Symptomatic and -Asymptomatic Roses in Central Georgia, USA

**DOI:** 10.3390/pathogens11020228

**Published:** 2022-02-09

**Authors:** Alejandra Monterrosa, Mathews L. Paret, Ronald Ochoa, Andrew Ulsamer, Shimat V. Joseph

**Affiliations:** 1 Department of Entomology, University of Georgia, 1109 Experiment Street, Griffin, GA 30223, USA; Alejandra.Monterrosa@uga.edu; 2 Plant Pathology Department, University of Florida, North Florida Research and Education Center, 155 Research Road, Quincy, FL 32351, USA; paret@ufl.edu; 3 Systematic Entomology Laboratory, USDA-ARS, Beltsville, MD 20705, USA; ron.ochoa@usda.gov (R.O.); andrew.ulsamer@usda.gov (A.U.)

**Keywords:** *Rosa* spp., eriophyid mite, *Emaravirus*, witches’ broom, phenology, rose bud mite

## Abstract

**Simple Summary:**

Rose rosette disease (RRD) is a serious disease of rose caused by the rose rosette virus (RRV). An eriophyid mite, *Phyllocoptes fructiphilus,* is the vector of RRV. The RRD symptoms affect the normal growth and development of rose plants. Because there is no cure for RRD, this disease threatens the rose industry, including container nurseries and cut flowers in the U.S. The seasonal occurrence and abundance of the vector and the locations they colonize on the plant are poorly studied in Georgia. The eriophyid mites are active from April to December on rose plants. The eriophyid mites were more abundant on the plants with RRD symptoms than on plants without any symptoms. The mites were found on both closed and opened flower buds alike. More mites were found on leaf bases and sepals than on other plant parts, such as leaf surfaces, stem, petals, anthers, stigma, and style. These results will help to develop integrated pest management strategies for the mite vector and reduce the spread of RRD.

**Abstract:**

*Phyllocoptes fructiphilus* Keifer (Acari: Eriophyidae) is the vector of rose rosette virus (RRV), which causes rose rosette disease (RRD) in North America. The RRD symptoms, such as witches’ broom, flower, and leaf deformation, disrupt the aesthetic appearance of plants and cause plant mortality. Because there is no cure for RRV, it is critical to manage the vector and reduce the spread of the virus. The information on the phenology of *P. fructiphilus* on rose plants is essential to develop management strategies and reduce its spread. Thus, the objectives of the study were to determine 1) the phenology of eriophyid mites (including *P. fructiphilus*) in central Georgia due to its widespread occurrence in the state and 2) the incidence of eriophyid mites on closed and opened flower buds and other plant parts. In central Georgia, eriophyid mites, including *P. fructiphilus* were active on both symptomatic and asymptomatic plants from April to December. The mite densities were greater during July and August than during the remaining months on asymptomatic plants. The mites were more abundant on the RRD-symptomatic than on the asymptomatic plants. Similar numbers of eriophyid mites were observed on closed and opened flower buds. Eriophyid mite densities were greater on sepals and leaf bases than on other plant parts.

## 1. Introduction

Rose rosette disease (RRD) is a devastating disease of rose (*Rosa* spp.) in the U.S. It is caused by a negative-stranded RNA virus, rose rosette virus (RRV), in the genus *Emaravirus* [1]. RRV is transmitted by an eriophyid mite, *Phyllocoptes fructiphilus* Keifer (Acari: Eriophyidae) [2]. *P. fructiphilus* is host-specific and is only known to transmit RRV in the genus *Rosa* spp. [3]. In the U.S., the rose industry is valued at ~$400 million USD per year [4], where roses are primarily sold as potted plants and cut flowers. The RRD symptoms on rose plants include witches’ broom, flower and leaf deformation, discoloration and strapping of leaves, increased density of thorns, reddening of stems, and die-back [3,5,6]. Once roses are infected with RRV, some symptoms of infection appear on the plants between 30 and 146 d after exposure [7], and RRD symptomatic rose plants perish within a few years. *P. fructiphilus* feeding does not cause any direct damage to the rose. Because there is neither an effective tactic to manage the vector *P. fructiphilus* nor RRV, to date, the rose industry in the U.S. is urgently seeking relevant biological information on the vector or RRV to reduce the spread of RRD.

A few species of eriophyid mites have been reported from roses [6,8,9] and *P. fructiphilus* has been implicated to transmit RRV [7]. *P. fructiphilus* are white or yellowish-white in color [10] with a fusiform, soft body [6,11]. Adults are between 140 and 170 µm in length and between 40 and 50 µm in width [6,11]. *P. fructiphilus* is native to North America and is common on multiflora (*Rosa multiflora* Thunb.) [12] and other rose species. To date, seven rose species demonstrate effective resistance to RRV or *P. fructiphilus* and suppressed the expression of the RRD symptoms [3]. 

The phenology of eriophyid mites was widely studied on many hosts to develop management strategies [13,14,15]. Previously, phenology of *P. fructiphilus* was studied on asymptomatic and symptomatic multiflora rose in Indiana, where a higher population of *P. fructiphilus* was recorded on symptomatic than on asymptomatic rose bushes [5]. However, little is known about the phenology of eriophyid mites, including *P. fructiphilus* on roses in the southeastern U.S. This information will help us to develop integrated pest management strategies to reduce the incidence and dispersal of eriophyid mites including *P. fructiphilus* and RRV. Solo et al. [9] showed that *P. fructiphilus*, *Eriophyes eremus* Druciarek, and Lewandowski and unknown eriophyid mites were detected from roses during the growing season in the southeastern U.S. The major objectives of this study were to determine: (1) the phenology of eriophyid mites (including *P. fructiphilus*) in central Georgia and (2) the incidence of eriophyid mites (including *P. fructiphilus*) on closed and opened flower buds and various plant parts. 

## 2. Materials and Methods

### 2.1. Sampling Site and Plant

In 2019 and 2020, rose terminals were sampled from the University of Georgia Research and Education Garden, Griffin, Georgia. The samples were collected from ‘Knock out’ roses (Rosa ‘Radtkopink’, Rosa ‘Radtko’), prickly rose (*Rosa acicularis* Lindl.), and red drift roses (Rosa ‘Meigalpio’). The rose plants sampled were from 3 to 15 years old and were from 0.6 m to 1.8 m tall. The rose plants were irrigated regularly and were pruned back during Jan–March. No insecticides were applied to the roses or surrounding plants in the garden. RRD symptoms were first observed on the selected roses in 2016. Before sampling began in 2019 and 2020, the selected rose bushes were thoroughly examined for the presence of RRD symptoms. The rose plants that showed symptoms were tagged, and the presence of RRV was confirmed using RT-qPCR [16]. Four rose bushes with RRD symptoms were tagged as symptomatic, and four other bushes with no distinct RRD symptoms were tagged as asymptomatic categories each year. These selected rose bushes were at least 10 m apart in the garden.

### 2.2. Experimental Design and Sample Collection

Eight terminal samples of 10 cm long each were sampled from previously tagged symptomatic and asymptomatic rose bushes. Four samples were completely closed, and four other samples were from completely opened flower bud terminals. Each rose bush served as the experimental unit. The experimental treatments (opened or closed bud status) were arranged in a randomized complete block design with four replications. Sixteen rose terminal samples were collected at biweekly intervals, eight from symptomatic and the remaining eight from asymptomatic plants. The terminal samples were individually bagged.

Sampling was conducted from January to December and from March to December in 2019 and 2020, respectively. In 2020, sample collection was disrupted because of COVID-19 work restrictions imposed by the University of Georgia. The numbers of eriophyid mites on the terminal samples were evaluated on the same day of collection or the next day. Those samples that were not evaluated on the same day were stored in the refrigerator at 4 °C. A 10 cm terminal rose sample consisted of a stem, three leaves, and a flower bud (opened or closed). The numbers of eriophyid mites present on various parts of the terminal shoot were quantified. The parts of the terminal shoot examined for eriophyid mites were the stem, petals, sepals, leaves (adaxial and abaxial leaf surfaces), leaf bases, style, stigma, and anthers under 40 × magnification of a dissecting microscope (Leica M125, Leica Microsystems, Wetzlar, Germany). 

### 2.3. Mite Identification

In 2019, the terminal rose samples (~10 cm long) were collected and sent to the North Florida Research and Extension Center, University of Florida in Tallahassee, Florida, USA. The rose samples were processed at the center based on the protocol described in Fife et al. [17] and Monfreda et al. [18]. The samples were soaked in 1:1 bleach: water and dishwasher detergent (0.2%) solution. After vigorous stirring, the solution passed through 180, 53, and 25 μm sieve in a decreasing order of mesh size. The 25 μm sieve screen has openings smaller than the average size of *P. fructiphilus*. Using a dissecting microscope, mites were siphoned off with a glass pipette into micro-centrifuge containers in 95% ethanol. A few mite specimens were slide mounted using Hoyer’s slide mounting media (Hempstead Halide, Inc. Galveston, Texas), and dried at 90 °C. The mites were identified at Florida Department of Agriculture and Consumer Services, Gainesville, Florida, USA. The character diagnostic of *P. fructiphilus* was the distinctive pattern of ridges on the prodorsal shield, among other characters [17,19]. 

In 2021, eriophyid mites (~40 mites) were individually collected from rose flowers using a paint brush under 40 × magnification of a dissecting microscope and were stored in ethanol (95%) in glass vials. The mite samples were sent to Systematic Entomology Laboratory, USDA-ARS, Beltsville, Maryland, USA, for identification. Specimens were examined using a Hitachi TM3030Plus tabletop scanning electron microscope (SEM) equipped with a Deben (UK) Coolstage. Multiple specimens were placed onto a sticky carbon pad, which was then mounted on to a steel viewing stub. Observations were made at −25 °C to reduce the amount of evaporation inside the microscope and extend observation time. *Phyllocoptes fructiphilis* can be recognized by its reticulate prodorsal shield, located on the anterior portion of the dorsum (top side of the body) and just posterior to the gnathosoma, having 10–12 irregular shaped raised rectangular cells and a median ridge starting at its anterior margin extending half way down the shield (Figure S1). The prodorsal shield has a frontal lobe, which partially covers the underlying gnathosoma (Figure S2). The female genital coverflap found on its venter (bottom side of the body) has five to nine longitudinal ridges, slightly directed medially (Figure S3). Above the genital coverflap there is a single ridge followed by eight short dotted ridges progressing towards the anterior part of the mite (Figure S4). The evaluation and comparison of the specimens as well the process to manipulate the material follow the techniques presented by Otero-Colina et al. [6]. 

Because not all the eriophyid mites were identified to species in the current study and possibility of co-occurrence of multiple species of eriophyid mites [6,8], the mites presented in the current study are, hereafter, referred to mostly as eriophyid mites in the following sections. The identified mite samples have been deposited in the Smithsonian Insect and Mite Collection located at the USDA-SEL as voucher specimens. 

### 2.4. Statistical Analyses

All statistical analyses were conducted in the SAS software [20]. The eriophyid mite data were arranged by sampling date and RRD symptomatic or asymptomatic status and were log-transformed (ln[x + 1]) after checking normality. The eriophyid mite data were subjected to two-way analysis of variance (ANOVA) using PROC GLM procedure in SAS. The effects of sampling date, RRD symptom status, and their interaction were included in the model. To understand the effects of the sampling date, eriophyid mite data were further analyzed by RRD symptom status as one-way ANOVA using PROC GLM procedure. The eight rose terminals were the replicates for this analysis.

To understand the incidence of eriophyid mites on closed and opened flower buds, and parts of the terminal shoot, eriophyid mite data were re-arranged by RRD symptom status where sampling date, the status of the buds (closed or opened), parts of shoot terminal (leaves, stem, sepals, etc.) and interaction between sampling date and specific location factors, such as closed or opened buds were the variables. Two-way ANOVA was performed on log-transformed (ln[x + 1]) eriophyid mite data using the PROC GLM procedure. To understand the effect of status of the buds (closed or opened) or parts of shoot terminal (leaves, stem, sepals, etc.) on eriophyid mites by sampling date, eriophyid mite data were further analyzed by RRD symptom status as one-way ANOVA using PROC GLM procedure. The means were separated using Tukey’s HSD (honestly significant difference) method at α = 0.05. Means and standard error for the variables were calculated using the PROC MEANS procedure in SAS.

## 3. Results

In June 2019, subsamples of eriophyid mites collected from symptomatic, and asymptomatic rose bushes were identified as *P. fructiphilus* using morphological characters described in the previous section. Similarly, all the eriophyid mites examined in 2021 were *P. fructiphilus*. Although all the eriophyid mites sampled for identification were identified as *P. fructiphilus* in 2019 and 2021 (Figures S1–S4) and there is still possibility of co-occurrence of multiple species of eriophyid mites in the same samples, the mites presented are not specifically referred to as *P. fructiphilus*.

### 3.1. Sampling Date and RRD Status

**Figure 1:**
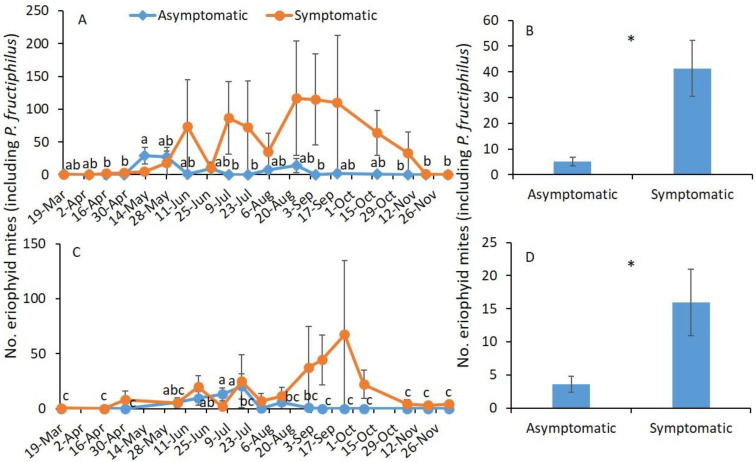
Mean (±SE) number of eriophyid mites (including *P. fructiphilus*) observed through the season in (**A**) 2019 and (**C**) 2020 and on plants with and without RRD symptoms in (**B**) 2019 and (**D**) 2020. The same letters among sampling dates or * on the bars between RRD symptomatic plants are not significantly different (ANOVA followed by Tukey’s HSD test; α = 0.05). Where no significant differences were observed, no letters or asterisks are included.

In 2019, sampling date and RRD status (asymptomatic or symptomatic) were significantly different, whereas their interaction was not significantly different (Table 1). To understand further, analysis was performed by RRD status, and the seasonal abundance of eriophyid mites was examined. A significantly greater number of eriophyid mites was collected on 13 May than on the remaining sampling dates except on 25 June on asymptomatic plants (*F*_17,51_ = 3.3; *p* = 0.001; Figure 1A), but on symptomatic plants, there was no significant difference in eriophyid mite densities between sampling dates (*F*_17,51_ = 1.3; *p* = 0.255). The numbers of eriophyid mite were significantly greater on symptomatic plants than on asymptomatic plants (Table 1; Figure 1B).

In 2020, only the RRD status significantly affected the number of eriophyid mites, whereas the sampling date and interaction between sampling date and RRD status did not significantly affect the number of eriophyid mites collected from the terminal shoots (Table 1). When examined by RRD status, a significantly greater number of eriophyid mites was observed on 5 July and 18 July than on the remaining sampling dates except on 5 June and 19 June on asymptomatic plants (*F*_15,49_ = 2.4; *p* = 0.011; Figure 1C). A similar number of eriophyid mites was found on 5 June, 19 June, 5 July, and 18 July. On symptomatic plants, there was no significant difference in number of eriophyid mites between sampling dates (*F*_15,49_ = 1.0; *p* = 429; Figure 1C). The number of eriophyid mites found was significantly greater on symptomatic plants than on asymptomatic plants (Table 1; Figure 1D).

### 3.2. Sampling Date and Closed or Opened Flower Buds

**Figure 2:**
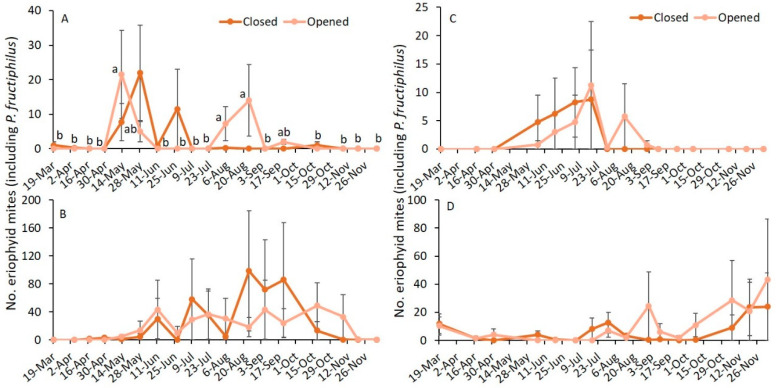
Mean (±SE) number of eriophyid mites (including *P. fructiphilus*) observed on open and closed flower buds through the growing season in 2019 (**A**) RRD asymptomatic and (**B**) RRD symptomatic and in 2020 (**C**) RRD asymptomatic and (**D**) RRD symptomatic plants. The same letters among sampling dates are not significantly different (ANOVA followed by Tukey’s HSD test; α = 0.05). Where no significant differences were observed, no letters are included.

In 2019, the effect of sampling date was significantly different, whereas the closed or opened status of the flower buds and interaction between sampling date and status of the buds were not significantly different on asymptomatic plants (Table 2). On symptomatic plants, sampling date, the status of the flower buds, and their interaction were not significantly different. When the analysis was performed to determine the effect of sampling date by RRD status and status of the flower buds, a significantly greater number of eriophyid mites was observed on 13 May, 5 August, and 24 August than on the remaining sampling dates on the opened flower buds (*F*_17,51_ = 2.6; *p* = 0.004; Figure 2A), whereas there was no significant difference in the number of eriophyid mites observed on the closed flower buds on asymptomatic plants (*F*_17,51_ = 1.6; *p* = 0.099). On symptomatic plants, numbers of eriophyid mites observed on the opened (*F*_17,51_ = 0.8; *p* = 0.637) and the closed flower buds (*F*_17,51_ = 0.8; *p* = 0.635; Figure 2B) were not significantly different by sampling date.

In 2020, the effect of sampling date, status of the flower buds, and their interaction were not significantly different on the numbers of eriophyid mites for asymptomatic and symptomatic plants (Table 2). When analysis was performed to determine the effect of sampling date by RRD status and status of the flower buds, the number of eriophyid mites was not significantly different on opened (asymptomatic: *F*_15,49_ = 0.5; *p* = 0.907; Figure 2C; symptomatic: *F*_15,49_ = 0.6; *p* = 0.824; Figure 2D) and closed flower buds (asymptomatic: *F*_15,49_ = 1.3; *p* = 0.263; Figure 2C; symptomatic: *F*_15,49_ = 1.1; *p* = 0.364; Figure 2D). 

**Figure 3:**
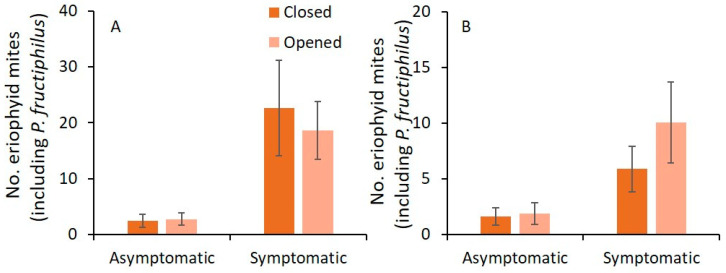
Mean (±SE) number of eriophyid mites (including *P. fructiphilus*) observed on closed and opened flower buds within RRD asymptomatic or symptomatic plants in (**A**) 2019 and (**B**) 2020. ANOVA followed by Tukey’s HSD test; α = 0.05 was performed. Where no significant differences were observed, no letters are included.

In 2019 and 2020, the numbers of eriophyid mites found on closed and opened flower buds for asymptomatic and symptomatic plants were not significantly different (Table 2; Figure 3).

### 3.3. Sampling Date and Terminal Plant Parts

**Figure 4:**
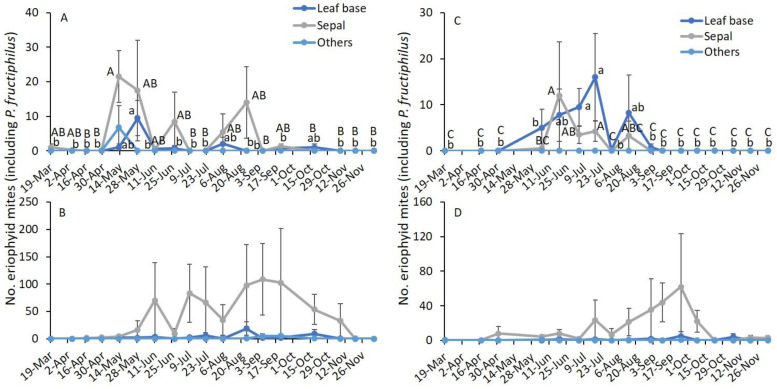
Mean (±SE) number of eriophyid mites (including *P. fructiphilus*) observed on terminal plant parts (leaf base, sepals, and other parts) through the growing season in 2019 (**A**) RRD asymptomatic and (**B**) RRD symptomatic, and in 2020 (**C**) RRD asymptomatic and (**D**) RRD symptomatic plants. The same letters among sampling dates are not significantly different (ANOVA followed by Tukey’s HSD test; α = 0.05). Where no significant differences were observed, no letters are included.

**Figure 5:**
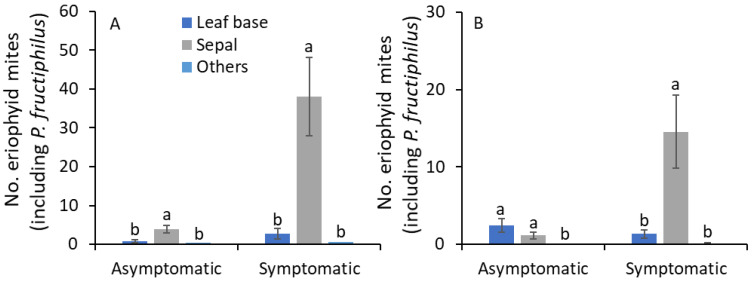
Mean (± SE) number of eriophyid mites (including *P. fructiphilus*) observed on terminal plant parts (leaf base, sepals, and other parts) on RRD asymptomatic or symptomatic plants in (**A**) 2019 and (**B**) 2020. The same letters within RRD asymptomatic or symptomatic plants for each year are not significantly different (ANOVA followed by Tukey’s HSD test; α = 0.05).

In 2019, sampling date and terminal plant parts and their interaction significantly affected the number of eriophyid mites on asymptomatic plants (Table 3). On symptomatic plants, sampling date and terminal plant parts significantly affected the number of eriophyid mites, but their interaction was not significantly affected. To understand this, further analysis was performed by RRD status and terminal plant parts to access the seasonal abundance of eriophyid mites. On the leaf base of asymptomatic plants, the number of eriophyid mites was significantly greater on 28 May than on the remaining sampling dates except for 13 May, 6 August, and 18 October (*F*_17,51_ = 2.6; *p* = 0.005; Figure 4A). On sepals, a significantly greater number of eriophyid mites was collected on 13 May than on 16 April, 1 May, 9 July, 22 July, 24 August, 6 September, 18 October, 8 November, 20 November, and 5 December (*F*_17,51_ = 2.5; *p* = 0.006). On other plant parts, there was no significant difference in eriophyid mite densities between sampling dates (*F*_17,51_ = 1.6; *p* = 0.095). The eriophyid mite densities found on symptomatic plants were not significantly different along the growing season on leaf bases (*F*_17,51_ = 1.4; *p* = 0.183; Figure 4B), sepals (*F*_17,51_ = 1.0; *p* = 0.436) and other plant parts (*F*_17,51_ = 1.6; *p* = 0.095). On asymptomatic and symptomatic plants, a significantly greater number of eriophyid mites was found on sepals than on leaf bases and all other plant parts combined (Table 3; Figure 4 and Figure 5A). 

In 2020, on asymptomatic plants, sampling date and terminal plant parts significantly affected the number of eriophyid mites, but their interaction was not significantly different (Table 3). On symptomatic plants, only terminal plant parts were significantly affected by the number of eriophyid mites. When the analysis was performed on RRD status and terminal plant parts, a significantly greater number of eriophyid mites was observed on the leaf base of asymptomatic plants on 5 July and 18 July than on the remaining sampling dates except on 19 June (*F*_16,48_ = 2.9; *p* = 0.025; Figure 4C). On sepals of asymptomatic plants, the number of eriophyid mites was significantly greater on 19 June and 18 July than on the remaining sampling dates except for 5 July and 14 August (*F*_16,48_ = 2.5; *p* = 0.008). On other plant parts, no eriophyid mite individuals were collected. The number of eriophyid mites found on symptomatic plants was not significantly different along the growing season on leaf bases (*F*_16,48_ = 1.1; *p* = 0.423; Figure 4D), sepals (*F*_16,48_ = 1.1; *p* = 0.358) and other plant parts (*F*_16,48_ = 0.9; *p* = 0.572). On asymptomatic plants, a significantly greater number of eriophyid mites was found on sepals and leaf bases than on all other plant parts (Table 3; Figure 5B). On symptomatic plants, the number of eriophyid mites was significantly greater on sepals than on leaf bases and other plant parts.

## 4. Discussion

The sub-samples of eriophyid mites collected from the roses were all identified as *P. fructiphilus* in the current study, although other eriophyid mites may occur on rose terminals [6,8]. *P. fructiphilus* is one of the major mite species reported in the USA other than the *E. eremus* [8] and recent report of *P. arcani* sp. nov [21] that are known to utilize floral habitat of roses [6]. In the current study, most of the mites were recovered from floral tissues, such as sepals and petals (92.2% [*n* = 3349] in 2019 and 80.6% [*n* = 1327] in 2020), and that mite densities were similar between opened and unopened flower buds (Table 3, Figure 2 and Figure 3). This result suggests that most of the eriophyid mites in the samples are likely *P. fructiphilus*.

The results showed that eriophyid mites were prevalent during the entire growing season, beginning mid-April to December in both years in central Georgia on roses. On asymptomatic plants, eriophyid mite densities spiked in May, July, and August more than in other summer or fall months. In 1987–1988, Amrine [5] found that *P. fructiphilus* densities also spiked from April to October on multiflora roses in Indiana. In the current study, because the variability of eriophyid mite numbers was unusually high, there was no significant difference in eriophyid mite densities collected throughout the sampling dates from symptomatic plants in both years. Previous studies showed that the RRD symptoms developed more slowly on shaded areas of multiflora rose plants than on full sun-exposed plants [22], and *P. fructiphilus* densities observed on shaded plants were lower than those observed on plants exposed to full sun [23]. High populations of eriophyid mites including *P. fructiphilus* on RRD-symptomatic plants in general landscapes threaten the ornamental rose industry, although *P. fructiphilus* is still the only species proven to act as a vector.

In the current study, *P. fructiphilus* densities were four to eight times more abundant on symptomatic than on asymptomatic plants, and these results were consistent with previous studies [5,6,22,23]. Although the causal factors of this variance are not known, there could be a few possibilities. First, the RRD symptomatic rose terminals demonstrate inconsistent and abrupt growth patterns, especially on the lateral shoots with strapped, red leaves, and partially-opened, small flower buds. It is possible that RRV-induced growth patterns alter the microhabitats of *P. fructiphilus* with access to additional refugia sites within the rosette terminals and provide more opportunities to develop into a large-sized population [23]. Second, it is possible that the RRV alters the behavior of *P. fructiphilus* individuals for its benefit, where dispersing mites prefer the RRD-symptomatic plants over asymptomatic plants [9]. Finally, *P. fructiphilus* may have the ability to detect the presence of RRV on the plants via specialized sensilla located on Legs 1 and 2 (Figures S1–S4) [24]. This suggests that more research is warranted to understand the underlying mechanism(s) driving the abundance of *P. fructiphilus* on RRD-symptomatic than on asymptomatic plants. 

Similar densities of eriophyid mites (including *P. fructiphilus*) were present on completely opened and completely closed flower buds throughout the growing season, regardless of the status of RRD symptoms. This is new information not reported previously and can have serious implications for *P. fructiphilus* management. In nurseries, RRD symptomatic plants are sporadically noted, and growers routinely apply miticides to mitigate the risk of mite population outbreak. When a risk of RRD is perceived in the facility, growers spray with miticides before shipping to wholesale, retail, or garden centers. Because eriophyid mites (including *P. fructiphilus*) were found in the closed flower buds, it is possible that *P. fructiphilus* were shielded from miticide applications. Most miticides used for eriophyid mites control function through contact rather that systemic action and ingestion. Incidence of *P. fructiphilus* on floral parts increases the dispersal risk of viruliferous *P. fructiphilus* through the cut flower trade [6,25]. Thus, more research is warranted to enhance *P. fructiphilus* exposure to miticides and prevent the spread of *P. fructiphilus* and RRV to nurseries. 

Eriophyid mites (including *P. fructiphilus*) densities were higher on the sepals of flower buds and leaf bases near flower buds than on any other areas of the rose parts, such as petals, male parts (stamen, anthers), female parts (stigma, style, etc.), abaxial and adaxial sides of leaves, bracts, and petioles in the current study. This result was consistent with Amrine [5] and Otero-Colina et al. [6]. Overwintering individuals of *P. fructiphilus* access the green tissues of bud scales on the rose stem [5]. In the spring, they move onto developing shoots on folded leaves [5], then colonize the leaf bases and sepals as these sites become available. The potential role of lower levels of feeding stimulants, such as carbohydrates or protein, and greater levels of feeding inhibitors, such as phenolic compounds, have been implicated as a limiting factor in *Epitrimerus gibbosus* (Nalepa) on blackberry leaves [26]. In the rose flower buds, the incidence of sugars is relatively high [27], and fluctuations in phenolic compounds were reported [28]. These studies suggest that more research is warranted to understand the population dynamics of *P. fructiphilus* with changes in flower development, levels of nutrients, and phenolics in the sepals and leaf base.

In summary, the results showed that eriophyid mites (including *P. fructiphilus*) were active from April to December in central Georgia, with population spikes during July and August on asymptomatic plants. The spikes in eriophyid mite populations on RRV-infected plants (and potential dispersal) seem of far greater importance than on asymptomatic or noninfected plants. Similar densities of eriophyid mites, including *P. fructiphilus*, were observed on the closed and opened flower buds. The *P. fructiphilus* densities were greater on leaf bases and sepals than on other plant structures. The information on the prevalence of eriophyid mites (including *P. fructiphilus*) regardless of flower bud status (open or closed) is new information not reported before. The results from the current study will shape the development of integrated pest management strategies for *P. fructiphilus* in ornamental landscapes and nurseries and this would perhaps include selection of miticides with systemic, rather than contact activity.

## Figures and Tables

**Table 1:** Two-way ANOVA for sampling date and RRD status on eriophyid mites (including *P. fructiphilus*).

Year	Treatment	*F*	df	*p*
2019				
	Sampling date	1.8	17,105	0.029
	RRD status *	13.3	1105	0.004
	Sampling date × RRD status	1.5	17,105	0.112
2020				
	Sampling date	1.5	15,101	0.106
	RRD status	9.9	1101	0.002
	Sampling date × RRD status	1.1	15,101	0.359

* Rose rosette disease asymptomatic and symptomatic plant.

**Table 2:** Two-way analysis of variance for sampling date and flower bud status by RRD on eriophyid mites (including *P. fructiphilus*).

Variables	2019			2020		
	*F*	df	*p*	*F*	df	*p*
Asymptomatic						
Sampling date	3.1	17,105	<0.001	1.5	15,101	0.109
Flower bud status	0.3	1105	0.573	0.0	1101	0.963
Sampling date × flower bud status	1.2	17,105	0.319	0.2	15,101	0.999
Symptomatic						
Sampling date	1.4	17,105	0.157	1.2	15,101	0.307
Flower bud status *	0.5	1105	0.499	0.0	1101	0.898
Sampling date × flower bud status	0.3	17,105	0.993	0.5	15,101	0.947

* completely closed or opened flower buds.

**Table 3:** Two-way analysis of variance for sampling date and terminal plant parts by RRD on eriophyid mites (including *P. fructiphilus*).

Variables	2019			2020		
	*F*	df	*p*	*F*	df	*p*
Asymptomatic						
Sampling date	4.4	17,159	<0.001	4.1	16,150	<0.001
Plant parts *	8.4	17,159	<0.001	7.2	2150	0.001
Sampling date × plant parts	1.5	34,159	0.047	1.4	32,150	0.098
Symptomatic						
Sampling date	2.3	17,159	0.004	1.1	16,150	0.362
Plant parts	18.5	17,159	<0.001	16.5	2150	<0.001
Sampling date × plant parts	0.6	34,159	0.941	0.9	32,150	0.567

* Sepals, leaf base, and others (stem, abaxial and adaxial leaf surface, petiole, petals, style, stigma, anthers).

## Data Availability

The identified mite samples have been deposited in the Smithsonian Insect and Mite Collection located at the USDA-SEL.

## Supplementary Materials

The following supporting information can be downloaded at: https://www.mdpi.com/article/10.3390/pathogens11020228/s1. Figure S1: Phyllocoptes fructiphilus, dorsum; Figure S2: Phyllocoptes fructiphilus, dorsal shield; Figure S3: Phyllocoptes fructiphilus, ventral female; Figure S4: Phyllocoptes fructiphilus, close up of ventral region.
