# Supplementary material for: Temporal Incidence of Eriophyid Mites on Rose Rosette Disease-Symptomatic and -Asymptomatic Roses in Central Georgia, USA
Source: Pathogens. 2022 Feb 9;11(2):228. doi: 10.3390/pathogens11020228 (PMC8875826; doi:10.3390/pathogens11020228)
Supplement: Supplementary file 1 [file pathogens-11-00228-s001.zip › pathogens-1510734-supplementary.pdf]

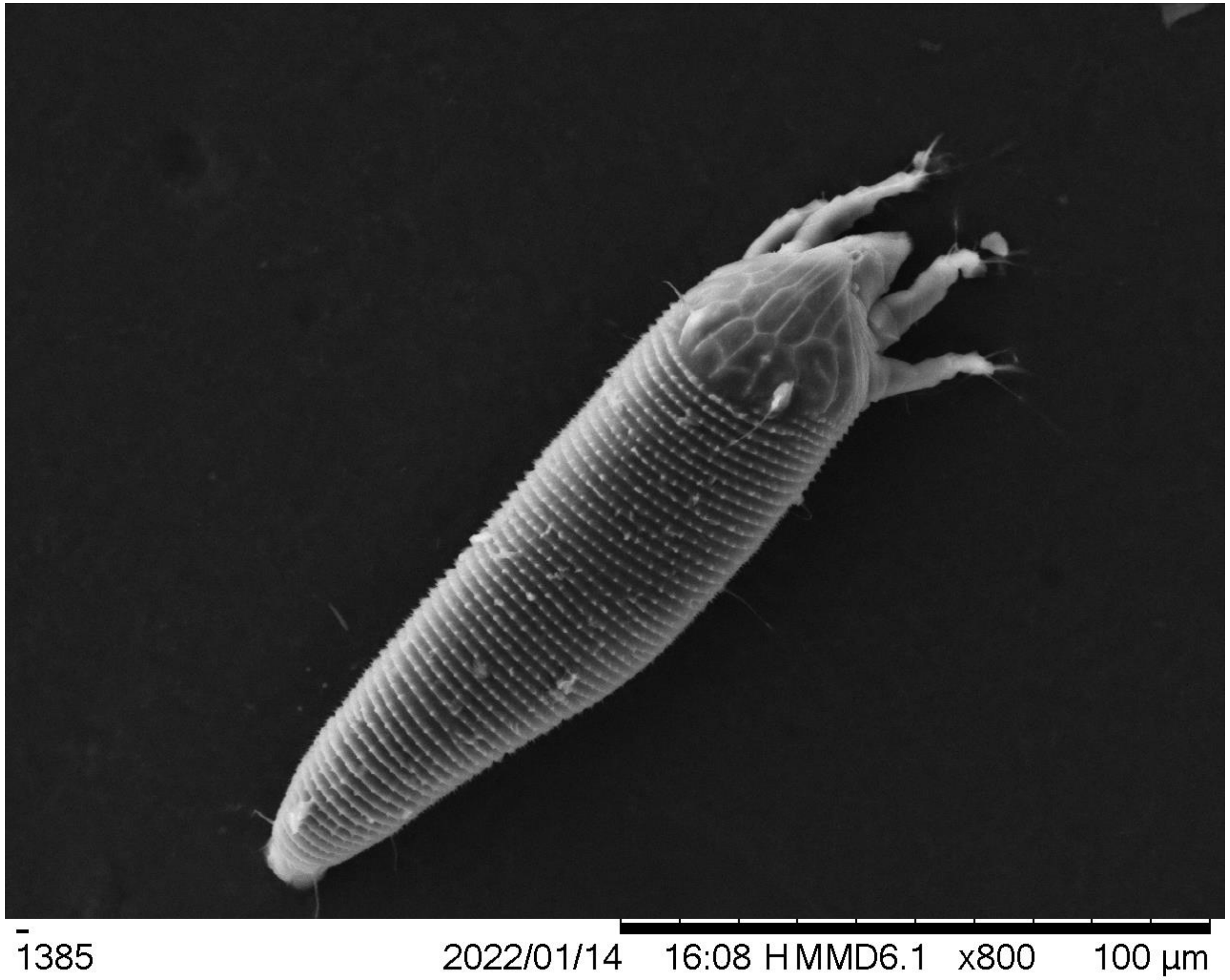

Figure S1: Phyllocoptes fructiphilus, dorsum

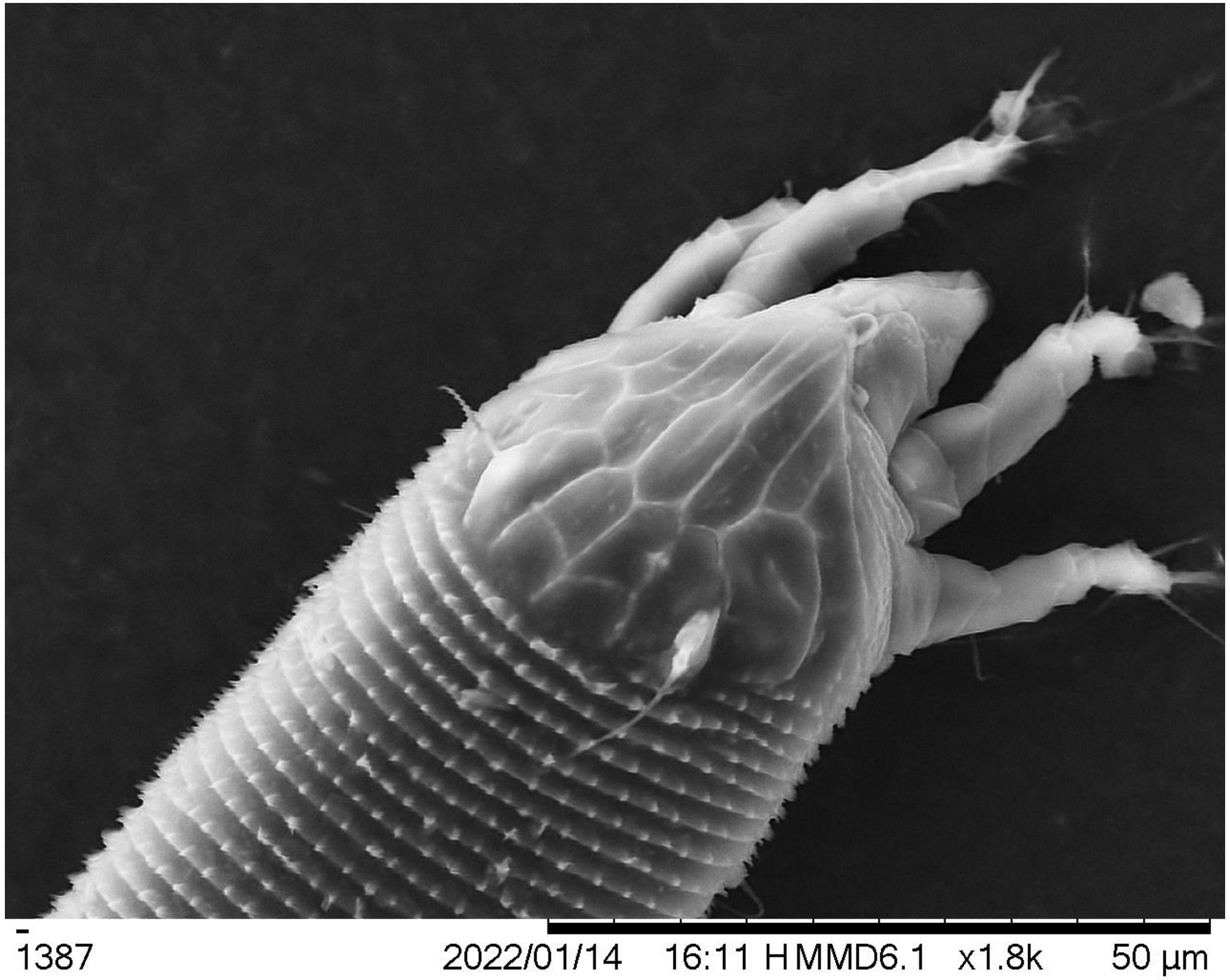

Figure S2: Phyllocoptes fructiphilus, dorsal shield

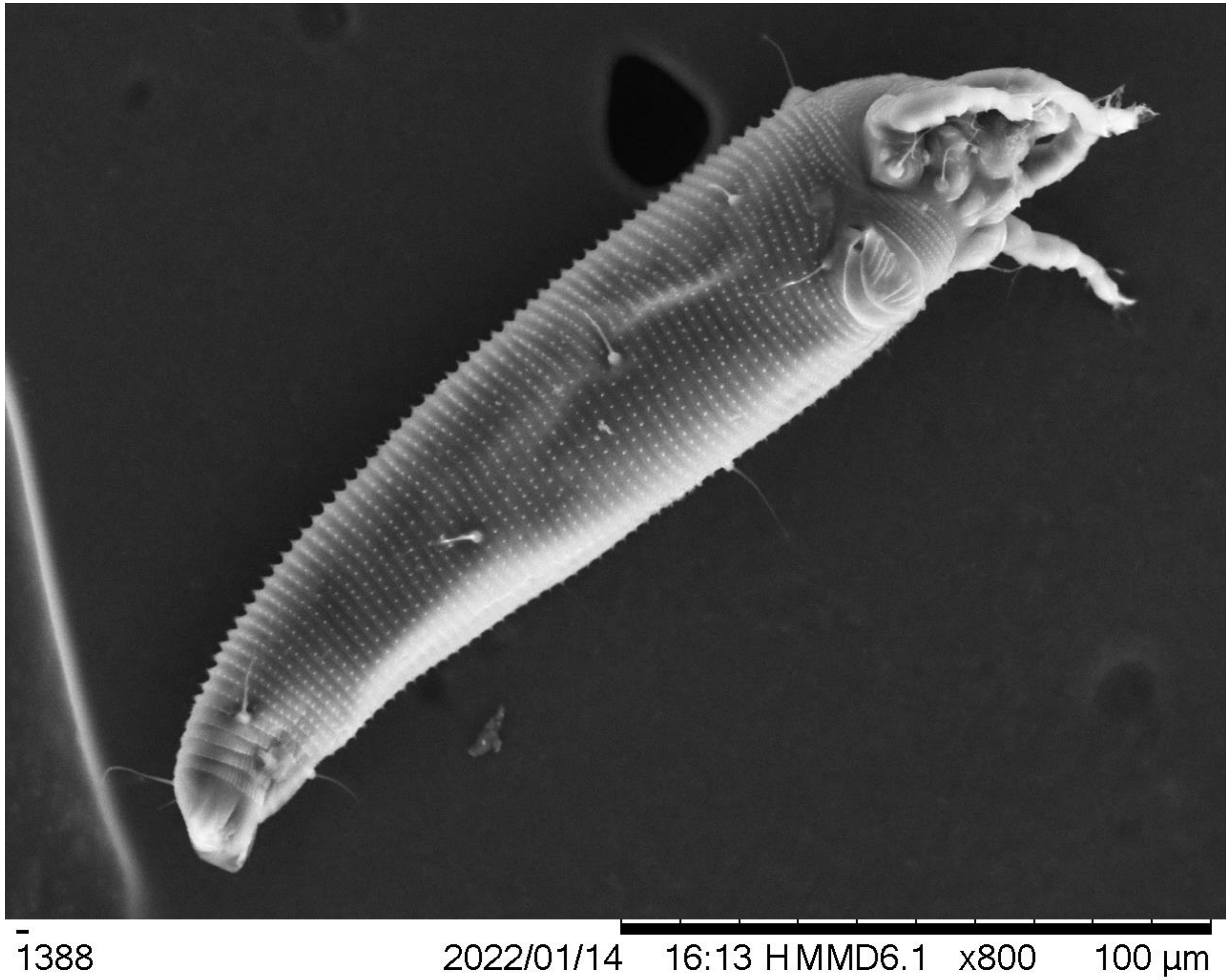

Figure S3: Phyllocoptes fructiphilus, ventral female

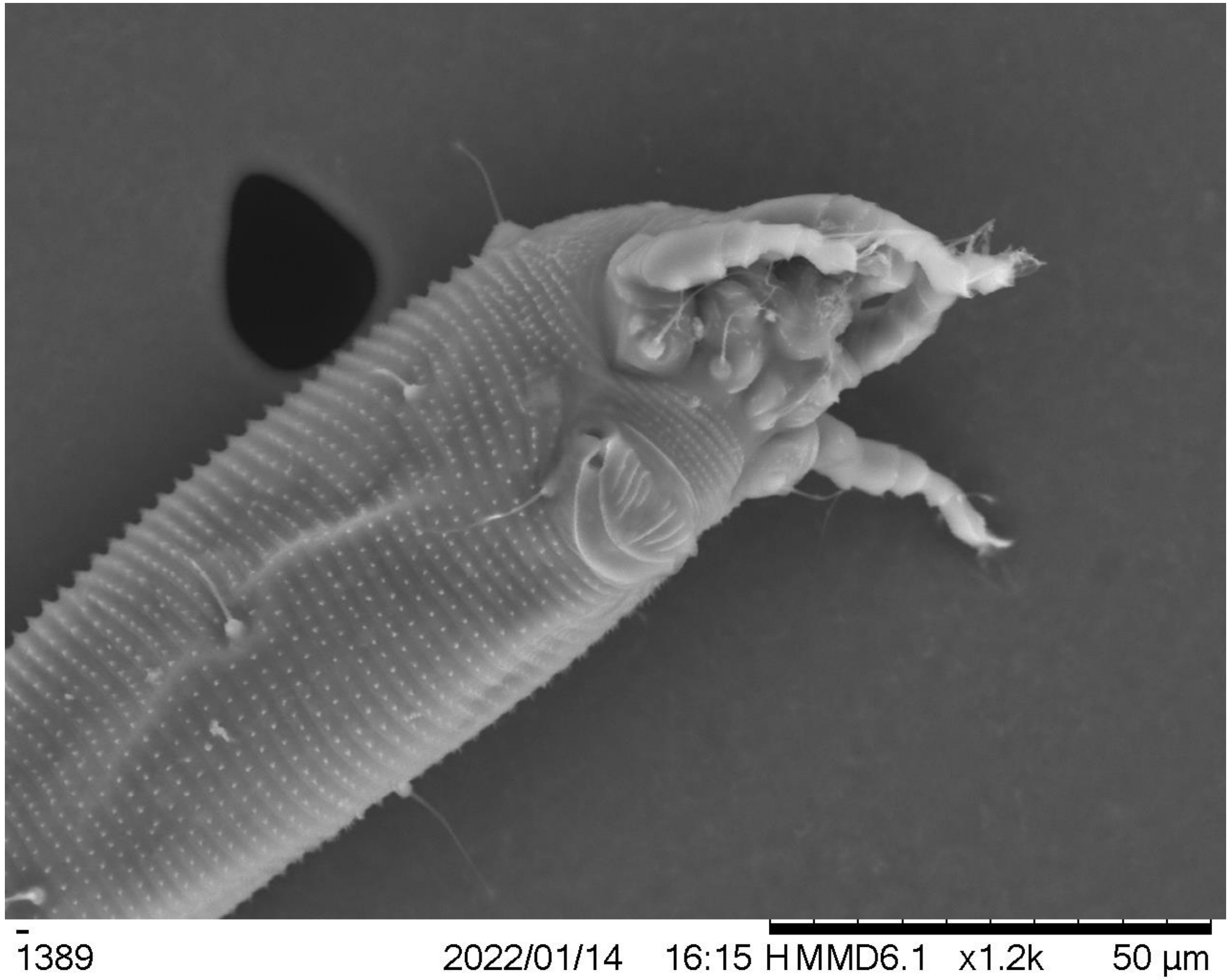

Figure S4: Phyllocoptes fructiphilus, close up of ventral region
